# Integrated analysis of genome-wide DNA methylation and cancer-associated fibroblasts identified prognostic biomarkers and immune checkpoint blockade in lower grade gliomas

**DOI:** 10.3389/fonc.2022.977251

**Published:** 2023-01-16

**Authors:** Jiawei Dong, Fang Wang, Xin Gao, Hongtao Zhao, Jiheng Zhang, Nan Wang, Zhihui Liu, Xiuwei Yan, Jiaqi Jin, Yixu Ba, Shuai Ma, Jianyang Du, Hang Ji, Shaoshan Hu

**Affiliations:** ^1^ Cancer Center, Department of Neurosurgery, Zhejiang Provincial People’s Hospital, Affiliated People’s Hospital, Hangzhou Medical College, Hangzhou, Zhejiang, China; ^2^ Department of Neurosurgery, The Second Affiliated Hospital of Harbin Medical University, Harbin, China

**Keywords:** DNA methylation, cancer-associated fibroblasts, lower-grade gliomas, prognosis, immune checkpoint blockade

## Abstract

**Background:**

Cancer-associated fibroblasts (CAFs) are vital components of prominent cellular components in lower-grade gliomas (LGGs) that contribute to LGGs’ progression, treatment resistance, and immunosuppression. Epigenetic modification and immunity have significant implications for tumorigenesis and development.

**Methods:**

We combined aberrant methylation and CAFs abundances to build a prognostic model and the impact on the biological properties of LGGs. Grouping based on the median CAFs abundances score of samples in the TCGA-LGGs dataset, differentially expressed genes and aberrantly methylated genes were combined for subsequent analysis.

**Results:**

We identified five differentially methylated and expressed genes (LAT32, SWAP70, GSAP, EMP3, and SLC2A10) and established a prognostic gene signature validated in the CGGA-LGGs dataset. Immunohistochemistry (IHC) and in vitro tests were performed to verify these expressions. The high-risk group increased in tumor-promoting immune cells and tumor mutational burden. Notably, risk stratification had different ICB sensitivities in LGGs, and there were also significant sensitivity differences for temozolomide and the other three novel chemotherapeutic agents.

**Conclusion:**

Our study reveals characteristics of CAFs in LGGs, refines the direct link between epigenetics and tumor stroma, and might provide clinical implications for guiding tailored anti-CAFs therapy in combination with immunotherapy for LGGs patients.

## 1 Introduction

Lower-grade gliomas (LGGs) including World Health Organization (WHO) grade II and III diffuse gliomas are slow-growing infiltrative brain tumors (1). Although the survival of LGGs patients after standardized treatment is better than that of glioblastoma (GBM), recurrent LGGs inevitably progress to GBM (2). With advances in the 2021 WHO Classification of Tumors of the Central nervous system, the understanding of molecular typing for glioma is gradually increasing. Exploration of epigenetics can help us better understand LGGs’ immunity and prognosis.

DNA methylation and gene expression are promising sources for identifying glioma’s molecular biomarkers. For instance, the promoter hypermethylation and epigenetic silencing of the O6-methylguanine-DNA methyltransferase (MGMT) gene have become a classical biomarker for temozolomide resistance glioma (3, 4). DNA demethylation and upregulation of IGF2BP3 can be involved in the malignant progression of glioma (5). Alternated DNA methylation in ZDHHC12 is associated with migration and invasion capabilities in glioma cell lines (6). GPX8 expression was correlated to the reduced DNA methylation at the promoter region and might be related to cancer-associated fibroblasts and immune infiltration levels in glioma (7). However, the clinical impact of these studies remains limited. Either due to the lack of drugs targeting these potential biomarkers or because of a breakthrough in immunotherapy.

Cancer-associated fibroblasts (CAFs) are the significant members of tumor stroma cells in the tumor microenvironment (TME) (8). Research on the significance of CAFs in cancer has recently gained momentum. Accumulating evidence has indicated that CAFs significantly affect tumor progression and migration, promote epithelial-mesenchymal transition (EMT), and induce chemoresistance and immunosuppression (9–12). On the other hand, CAFs and extracellular matrices constitute the tumor immune escape initiation mechanism (13). In response to this problem, harnessing CAFs-related immunosuppressive stromal environment has been proposed to ameliorate the response to immune checkpoint inhibitors (14, 15). However, whether CAFs are associated with the predictive value and immunotherapy of LGGs patients has not been elucidated.

We reasoned that LGGs samples with different CAFs scores broadly alter methylation levels and immune infiltration patterns. To verify the conjecture, we used a median of CAFs score as the grouping basis for the sample to gather genome-wide methylation and gene expression data to locate the altered methylations coupled with altered expression of the same genes. Then we constructed a risk score system containing five risk genes and validated them at tissue-level and cell-level. We found the risk score is an excellent predictive value for survival and a potential factor for immune checkpoint blockade (ICB) therapies. Applying this prognostic gene signature, the sensitivity of GDC0941, Bleomycin, and Axitinib showed a significant difference in sensitivity within the subgroups. These drugs may have different effects on patients with different levels of CAFs infiltration.

## 2 Material and methods

### 2.1 Data acquisition

**Figure 1:**
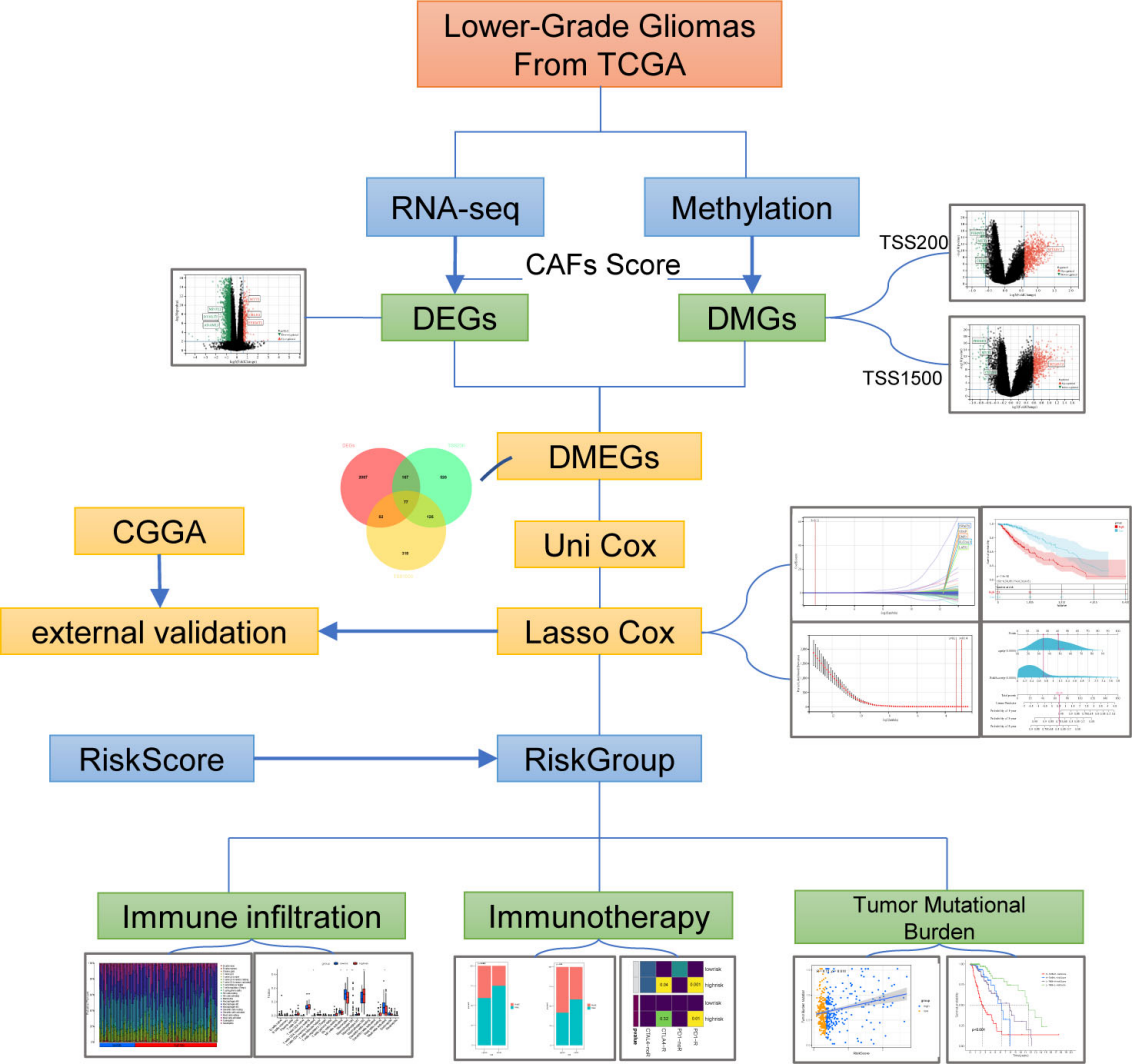
Study Flow Chart.

RNA-seq data and clinical data on LGGs were extracted from The Cancer Genome Atlas (TCGA, https://portal.gdc.cancer.gov/) and Chinese Glioma Genome Atlas (CGGA, http://www.cgga.org.cn/). After transcripts per million (TPM) conversion, we held genes with expression levels larger than 0.1 TPM for analysis. Matched Normal data: 105 cortex tissues were obtained from the GTEx project (https://commonfund.nih.gov/GTEx/). We downloaded both methylation data (Illumina Infinium HumanMethylation450 BeadChip) and somatic mutation data generated by TCGA from the UCSC Xena browser (https://xenabrowser.net/hub/) (16). Fibroblast and glioma cell lines from The Cancer Cell Line Encyclopedia (CCLE) (https://portals.broadinstitute.org/ccle) (17). Infiltration Estimation for TCGA-LGGs was collected from TIMER2.0 (http://timer.comp-genomics.org/). The STRING (https://www.string-db.org) database produced the protein-protein interaction network and reconstructed them *via* Cytoscape software. A flowchart of the entire procedure can be shown in **Figure 1**.

### 2.3 Analysis of DNA methylation data

The Illumina HumanMethylation450 BeadChip array contains probes covering 99% of reference sequence genes and 96% of CpG islands. The raw methylation intensities for each probe were represented as β-values, which were converted into M-values with R package Lumi for statistics analysis (18). 5’-C-phosphate-G-3’ (CpG) methylation data between different groups were compared with R package limma to identify differentially methylated CpG sites. Benjamini-Hochberg (BH) method was used to adjust p-value as a false discovery rate (FDR). The CpG site and gene mapping files were downloaded from illumine (https://www.illumina.com/). CpGs span various gene regions, including 1500 bp and 200 bp upstream of the transcription start sites (TSS1500 and TSS200, respectively). The average β-values for each region were calculated according to all CpG sites at the corresponding region, and the average β-value was converted to an M-value. Average regional methylation data between different groups were compared with R package limma to identify differentially methylated regions (DMRs). The hypermethylated DMRs with a threshold of adjusted p-value< 0.05 combined a delta β-value > 0.2, and the hypomethylated DMRs with a threshold of adjusted p-value< 0.05 combined a delta β-value< −0.2. Genes harboring DMRs in any part of the gene features were differentially methylated genes (DMGs). The equations described above are listed below.


(1) $$M_{i}=\log_{2}\left(\frac{\beta_{i}}{1-\beta_{i}}\right)$$

(2) $$\;\beta_{region}=2^{\left(\sum_{i=1}^{k}\log2\left(\beta_{i}\right)\right)∕k}$$

where M is the intensity of the methylated allele, i = 1, 2, 3,…, k, and k is the number of CpG sites in a region.

### 2.4 Cancer-associated fibroblasts (CAFs) infiltration estimation and immune score calculation

CAFs abundances were separately estimated *via* Estimate the Proportion of Immune and Cancer cells (EPIC) algorithm using the R package Immunedeconv (19). TCGA-LGGs were divided into a high-CAFs-score group and a low-CAFs-score group according to the median score. The estimated immune and stromal scores were computed using the R package ESTIMATE.

### 2.5 Analysis of DEGs and DMGs

Differential expression between the high-CAFs-score and low-CAF-score group samples was analyzed with the R package limma. False discovery rate (FDR) as adjusted p-value using the Benjamini-Hochberg (BH) method. The fold change was log2-transformed. Differentially expressed genes (DEGs) were calculated with a difference > 1.5-fold and p< 0.01. Methylation analysis results were carried out for joint analysis. Venn diagram analyses were performed to calculate the intersection of DMGs and DEGs and explored the differentially methylated and expressed genes (DMEGs). DMEGs were grouped according to four expression patterns: HypoUp, HypoDown, HyperUp, and HyperDown.

### 2.6 Functional enrichment analyses

To functionally annotate DMEGs of this study, Gene Ontology (GO) including biological process (BP), cellular component (CC), and molecular function (MF) analysis was performed in the R package ClusterProfiler (20). A p-value of< 0.05 and an FDR of< 0.05 were used for the cutoff value. The ClueGO Plugin version 2.5.8 in Cytoscape Version 3.8.2 was employed to identify hub genes and functional analysis (21).

### 2.7 Construction and validation of the risk score system

We selected HypoDown and HyperUp genes in TSS200 and TSS1500 for univariate cox regression analysis and filtrated the prognostic-related genes. Subsequently, we used the R package glmnet to conduct the least absolute shrinkage and selection operator (LASSO) Cox regression algorithm and develop a potential risk signature. The minimum value of lambda was derived from 1,000 cross-validations (‘1-se’ lambda), and which corresponding partial likelihood deviance value was the smallest for the risk model. Coefficients with regression were confirmed by the “cvfit” function with 1000 repeats. The risk score calculating equation, which contains five risk genes, is:


(3) $$R\text{i}skscor\text{e}=\sum_{i=1}^{n}Co\text{e}f_{\text{i}}*x_{i}$$

where Coe*f*_i_ means the coefficients, *x*_i_ is the expression value of each gene.

The predictive power of the prognostic signature was evaluated by the receiver-operating characteristic (ROC) curve. The independent clinical factor was validated by multivariate Cox regression analysis. Finally, a nomogram was constructed according to independent predictors. The calibration of the nomogram was evaluated by the calibration curve to assess the goodness of 1-, 3-, and 5-year overall survival.

### 2.8 Analysis of immunological characteristics

In this study, the mRNA expression matrix of LGGs was analyzed using the CIBERSORT R script downloaded from http://cibersort.stanford.edu. Based on deconvolution, we estimated the abundance of immune cell populations. The relationship between each immune cell and survival was measured by Kaplan-Meier (KM) survival analysis. We evaluated a total of 60 immune checkpoints (ICP) genes in two categories (Inhibitory ICP (22) and Stimulatory ICP (23)) from widely recognized literature (24). Then we assessed the expression and survival of these ICP in the TCGA-LGGs cohort for a comprehensive overview of the immunosuppressive landscape.

The Tumor Immunophenotype Profiling (TIP) was performed to quantify the extent of infiltrating immune cells and anticancer immunity (25). Assessment of antitumor immunity was conceptually divided into seven steps, including tumor cell antigen release (step 1), cancer antigen presentation (step 2), priming and activation (step 3), trafficking of immune cells to tumors (step 4), infiltration of immune cells (step 5), T cell recognition of cancer cells (step 6), and killing of cancer cells (step 7).

TIDE (http://tide.dfci.harvard.edu/), an excellent algorithm, was used to explore the prediction of clinical response to immune checkpoint blockade (ICB) therapy (22). The TIDE score was calculated to simulate two mechanisms of tumor immune evasion: the induction of T cell dysfunction with high infiltration of cytotoxic T lymphocytes and the retard of T cell infiltration in tumors with low cytotoxic T lymphocyte infiltration. The TIDE score is a good reflection of the responsiveness of the ICB. The SubMap (https://www.genepattern.org/) was carried out to validate the reliability of the prediction of TIDE.

The R package pRRophetic was used to predict chemotherapeutic response in LGGs patients (26). In addition to temozolomide, which patients with glioma widely used, we included three drugs with the therapeutic potential for glioma in this study: Axitinib, GDC-0941 (PI3K inhibitor), and Bleomycin.

### 2.9 Verification of gene expression at cellular level and tissue level

H4, SW1783, and HMF cell lines were purchased from ATCC. According to the manufacturer’s instructions, total RNA was isolated using Trizol reagent (Invitrogen, USA). 2μg of the total RNA was transcribed into cDNA. SYBR Green PCR kit (Takara, Japan) was used for qRT-PCR. We selected the 2−ΔΔCq method to calculate gene transcription level, with β-actin mRNA as control. Data represent the mean ± SD of triplicate real-time PCR. The primers were synthesized by Tsingke Biotechnology (Shanghai, China) and displayed in **Supplementary Table S1**. Immunohistochemistry (IHC) analyzed the protein expression levels. GSAP (ab106630), LATS2 (ab111054), SWAP70 (ab228846), and SLC2A10 (ab110528) antibody was purchased from Abcam. EMP3 (sc-81797) antibody was purchased from Santa Cruz Biotechnology. Clinical characteristics of LGGs patients cohort are displayed in **Supplementary Table S2**. All the patients and the hospital’s Ethics Committee approved this research. LGGs tissues were formalin-fixed, paraffin-embedded, and sectioned at 4 µm. Immune complexes were detected with the SP Kit (Solarbio, Beijing, China) and DAB Substrate Kit (Solarbio, Beijing, China). Signals were detected using an Olympus BX41 microscope. Quantification of Immunohistochemistry (IHC) staining was performed in a blinded fashion.

### 2.10 Statistical analysis

All the data were analyzed using the R software version 4.1.0. The overall survival (OS) between different groups was analyzed using Kaplan-Meier curves. Kruskal–Wallis tests were applied to compare gene expression in two groups. The Fisher test assessed different groups’ responses to ICB treatment. Somatic mutation data sorted in the form of Mutation Annotation Format (MAF) was analyzed using the R package maftools. ImageJ and ImageJ plugin IHC profiler was applied to quantify IHC staining analysis. IHC scoring data and qRT-PCR data were analyzed using GraphPad/Prism 9.0. In addition, tumor mutational burden (TMB) and mutation counts were computed from somatic mutation frequencies. p< 0.05 was marked as ‘*’, p< 0.01 was marked as ‘**’, p< 0.001 was marked as ‘***’. and p< 0.0001 was marked as ‘****’.

## 3 Results

### 3.1 Differentially methylated and expressed genes (DMEGs) in LGG

**Figure 2:**
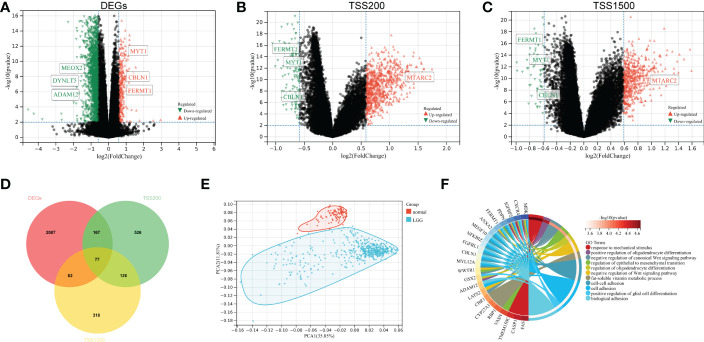
Identification and functional enrichment of DMEGs. **(A)** Volcano plot presenting the DEGs **(**difference > 1.5-fold, p-value< 0.01**)** between two groups. **(B)** DMGs **(**difference > 1.5-fold, p-value< 0.01**)** volcano plot of TSS200 region. **(C)** DMGs **(**difference > 1.5-fold, p-value< 0.01**)** volcano plot of TSS1500 region. **(D)** Venn diagram exhibiting DMEGs expressed in the TCGA dataset. **(E)** The profiles of DMEGs and principal component analysis **(**PCA**)** between tumor and normal cortex tissues. **(F)** GO analysis shows significant GO terms in DMEGs.

To identify DMEGs in LGGs, we first extracted the gene expression and DNA methylation data of TCGA-LGGs and performed a comparative analysis. Samples were divided into high- and low-CAFs-score groups according to the median CAFs score. From the summary estimate, 2393 statistically significant Differentially Expressed Genes (DEGs) were identified (difference > 1.5-fold, p-value< 0.01), including 263 upregulated and 2131 downregulated genes (**Figure 2A**, **Table S3**). Promoter regions (TSS200 and TSS1500) were enrolled in the primary study. As shown in volcano plots, 1276 DMGs were identified from two regions, including 896 DMGs in the TSS200 region (**Figure 2B**, **Table S4**) and 583 DMGs in the TSS1500 region (**Figure 2C**, **Table S5**).

We analyzed the relationship between methylation and gene expression by integrating DMGs and DEGs in two promoter regions (TSS1500 and TSS200). A total of 77 DMEGs were identified (**Figure 2D**), and then we performed a principal component analysis (PCA) of the DMEGs in normal tissue and LGGs (**Figure 2E**). The PCA profile revealed a clear separation of normal samples from LGGs. Afterwards, GO enrichment analysis was performed on these DMEGs, and the result indicated that DMEGs were significantly enriched in positive regulation of oligodendrocyte differentiation, negative regulation of canonical Wnt signaling pathway, and regulation of epithelial to mesenchymal transition (**Figure 2F**). These functions were closely related to the oncogenesis and progression of glioma.

### 3.2 DMEGs analysis in two promoter regions

**Figure 3:**
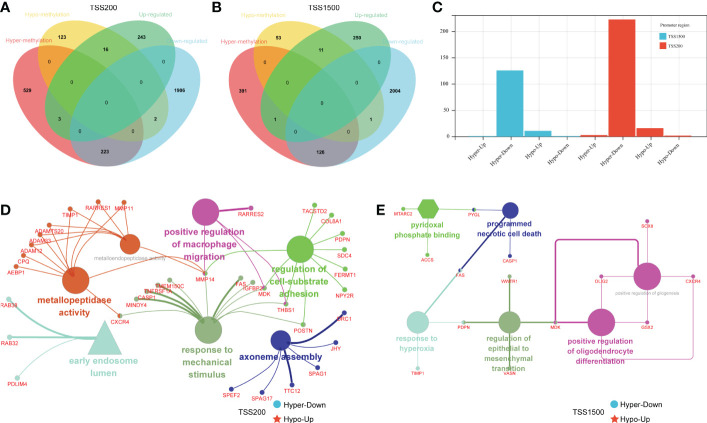
Grouping and functional analysis of DMEGs. **(A, B)** Venn diagram showed four different groups **(**HypoDown, HyperUp, and HyperDown**)** of DMEGs in the TSS200 region **(A)** and TSS1500 region **(B)**. **(C)** The bar chart shows the different groups of DMEGs in the two regions. **(D, E)** ClueGO Cytoscape network of statistically DMEGs in two regions.

To investigate the differences in DMEGs within each region, we classified these DMEGs into four groups (HypoUp, HyperUp, HyperDown, and HypoDown) for TSS200 and TSS1500, respectively (**Figures 3A, B** **)**. The HyperDown group was the most prevalent in TSS200 and TSS1500 regions, and the HypoUp group had the second-highest proportion in the two regions (**Figure 3C**). After extracting the HyperDown and HypoUp DMEGs in TSS200 and TSS1500 regions separately, we used STRING to construct PPI networks. These genes were analyzed by GO enrichment using the “ClueGO” plugin for Cytoscape software (p< 0.01, Kappa score = 0.5). Functional enrichment analysis revealed that these DMEGs participated in critical biological processes. In the TSS200 region, DMEGs are mainly associated with response to mechanical stimulus, regulation of cell-substrate adhesion, and positive regulation of macrophage migration (**Figure 3D**). As shown in **Figure 3D**, MMP14 is critically involved in various immune-related functions. Meanwhile, DMEGs in the TSS1500 region are mainly related to regulating epithelial to mesenchymal transition, programmed necrotic cell death, and positive regulation of gliogenesis (**Figure 3E**). These simultaneous differential DEGs and DMGs pooled to DMEGs may be the main factor causing the altered biological function of LGGs.

### 3.3 Construct the DMEGs prognostic signature

**Figure 4:**
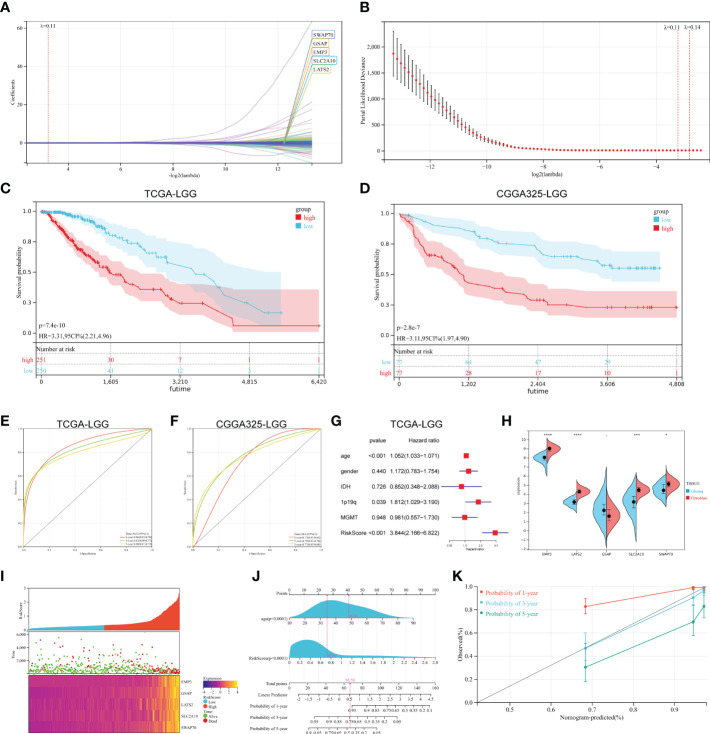
Construction of prognostic signature. **(A, B)** The process of building the signature. The least absolute shrinkage and selection operator **(**LASSO**)** regression was performed, calculating the minimum criteria. **(C, D)** K-M curves showed that the high-risk subgroup had worse overall survival than the subgroup in the train set **(**p< 0.001**)** and test set **(**p< 0.001**)**. **(E, F)** ROC curves showed the predictive efficiency of the risk signature on the 1-, 3- and 5-year survival rates of train set **(E)** and test set **(F)**. **(G)** Independent prognostic factors were determined by the multivariate Cox regression analyses. **(H)** Expression of five risk genes in fibroblast cell lines and glioma cell lines in the CCLE dataset. **(I)** The heatmap was based on the expression of the five genes in the high- and low-risk group. **(J, K)** A nomogram **(J)** and decision curve analysis **(K)** of the risk score for predicting 1-, 3- and 5-year survival. (*p < 0.05, **p <0.01, ***p <0.001, ****p <0.0001).

Among the DMEGs in HyperDown and HypoUp groups, Univariate Cox regression analysis screened 203 DMEGs with prognostic values. Then a Lasso‐penalized Cox analysis was performed to shrink further the scope of DMEGs screening (**Figure 4A**) and lambda. Min was regarded as the optimal value in the cross-validation process (**Figure 4B**). Five DMEGs (GSAP, EMP3, LATS2, SWAP70, and SLC2A10) and corresponding coefficients (**Table S6**) were identified. We used TCGA-LGGs as the train set CGGA-325 and CGGA-693 as the test sets, and samples were split into high- and low-risk groups by the median value of the risk score. KM survival curves depicted that LGG patients with increased risk scores had worse clinical outcomes in both train set and test sets (**Figures 4C, D**, and **Figure S1A**). Statistical analysis was performed using a log-rank test (train set p< 0.001, test set p< 0.001). After that, we established ROC curves of the risk score model with 1-year, 3-year, and 5-year. The results revealed that the risk score could effectively distinguish LGGs patients with different survival statuses (**Figure 4E** 1-yer AUC = 0.86, 3-year AUC = 0.83, and 5-year AUC = 0.80). The results were similar and slightly lower in the test set (**Figure 4F** 1-yer AUC = 0.73, 3-year AUC = 0.79, and 5-year AUC = 0.77). Multivariate Cox regression analysis showed the independent prognostic value of this risk score (**Figure 4G**, p< 0.001, HR = 3.844). Then we examined the mRNA expression of five risk genes in the glioma cell lines and fibroblast cell lines with CCLE (**Figure 4H**). Although glioma lines are not representative of LGGs, we found the expression of GSAP was consistent with that of fibroblasts, and the expression of the other four risk genes was lower than that of fibroblasts in glioma cell lines. The mortality of patients increased with the increase in the risk score (**Figure 4I**). Finally, we established the Nomogram model, which contained risk score and age to assess the survival prediction in LGGs patients (**Figure 4J**) and calibration curves for nomogram predicted 1-, 3- and 5-year overall survival performed well with the risk model (**Figure 4K**).

### 3.4 Correlation between risk score and clinical characteristics

**Figure 5:**
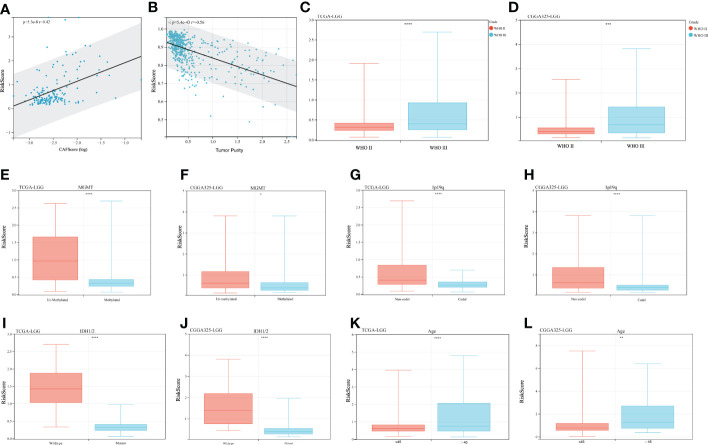
Clinical characteristics and risk scores. **(A)** Correlation of risk scores with CAFs scores, r = 0.42 p< 0.001. **(B)** Correlation of risk scores with tumor purity, r = -0.56, p< 0.001. **(C, D)** Risk scores for different WHO-graded samples, **(E, F)** MGMT promoter status, **(G, H)** 1p19q codeletion status, **(I, J)** IDH mutation status, and **(K, L)** Age effect. (*p < 0.05, **p <0.01, ***p <0.001, ****p <0.0001).

Clinical variables were introduced into the risk score system to analyze the relationship between risk scores and clinical characteristics. We initially compared the CAFs’ scores concerning the risk score. As shown in **Figure 5A**, the risk score was mildly positively associated with the CAFs score (r = 0.42, p< 0.01). In contrast, tumor purity was negatively correlated with the risk score (**Figure 5B** r = -0.56, p < 0.01). Other clinical features were then introduced. WHO grade III LGGs have a higher risk score than WHO grade II LGGs, which is consistent with their poor prognosis (**Figures 5C, D**, **Figure S1B**). The risk score in MGMT promoter methylated LGGs were significantly lower than that in MGMT promoter unmethylated LGGs samples (**Figures 5E, F**, **Figure S1C**). It is widely accepted that glioblastoma patients with MGMT promoter methylated are sensitive to temozolomide and suitable for TMZ chemotherapy. For another crucial molecular marker 1p19q codeletion status, the risk score was significantly higher in 1p19q-non-codeletion samples compared to codeletion samples in both datasets (**Figures 5G, H**, **Figure S1D**). Correspondingly, IDH1/2 wild-type cases showed a valid increased risk score compared to IDH1/2 mutant cases **(** **Figures 5I, J**, **Figure S1E**). The high-risk scores were seen in cases aged > 45 years. (**Figures 5K, L**, **Figure S1F** **)**. Overall, some important molecular markers and clinical features of LGGs responded well to this risk score system.

### 3.5 Prognostic signature and immune landscape

**Figure 6:**
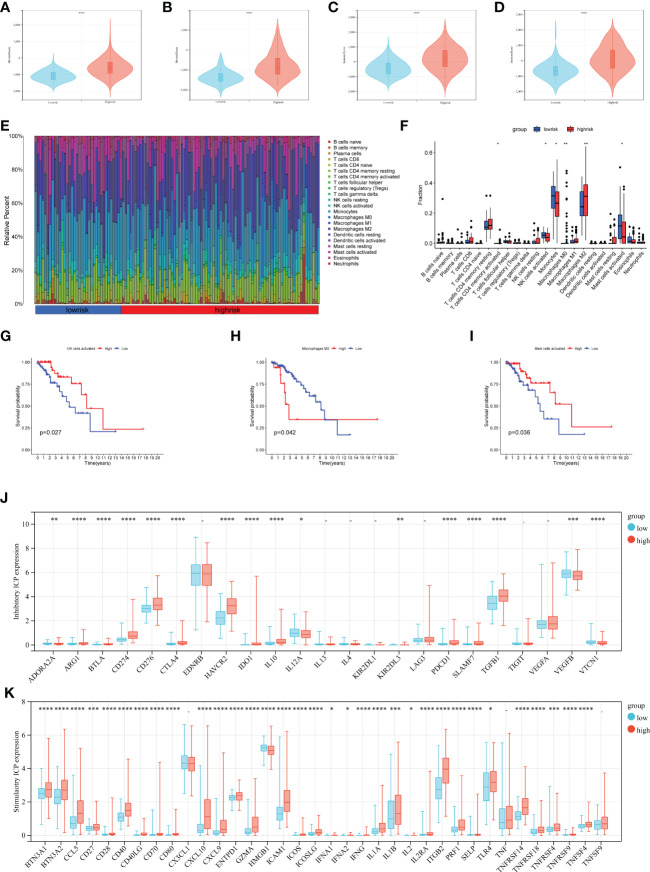
Analysis of immune infiltration in different risk groups. **(A–D)** Immune score and stromal score calculated by ESTIMATE in different groups **(A, C** TCGA data set, and **B, D** CGGA data set**)**. **(E, F)** The relative infiltrating proportion of 22 immune cells in high- and low-risk groups. **(G–I)** KM curves of infiltrating immune cells associated with survival in LGGs patients (p< 0.05). **(J, K)** Immune checkpoints expression in the LGGs microenvironment, inhibitory ICP **(J)**, and stimulatory ICP **(K)**. (*p < 0.05, **p <0.01, ***p <0.001, ****p <0.0001).

To investigate the relationship between the prognostic signature and immune cell infiltration in LGGs. We evaluated immune scores, immune cell infiltration, and immune checkpoints separately. CAFs are the most prominent tumor stroma cell type in the TME. Comparing the stromal score and immune score of the LGGs datasets, we found a significantly positive correlation between the risk score and stromal score, and the same was true of the immune score (**Figures 6A, B**, stromal score p< 0.001 and **Figures 6C, D** immune score p< 0.001). CIBERSORT algorithm showed the proportions of distinct immune cell subpopulations in different risk groups (**Figure 6E**). The relative expression is shown in bar diagrams. The proportions of Macrophages M0 and Macrophages M2 in the high-risk group were significantly higher than in the low-risk group (**Figure 6F**). By contrast, the proportions of Monocytes, activated NK-cell and activated Mast-cells were higher in the low-risk group. Subsequently, we performed KM survival analysis to evaluate the OS with differing immunocytes infiltration samples (**Figures 6G–I**). High proportions of activated NK-cell and activated Mast-cell had a better OS (**Figures 6G, I** **)**. Conversely, the level of macrophage M0 expression is inversely related to OS (**Figure 6H**). We next examined whether the risk score is associated with the expression of inhibitory and stimulatory immune checkpoint (ICP) molecules. Fortunately, the expression of many immune checkpoint molecules showed significant differences between high and low-risk groups (**Figure 6J** Inhibitory ICP and **Figure 6K** stimulatory ICP). The KM curves for survival analysis of these immune checkpoints were presented in **Figure S2**. In summary, the risk score was associated with the expression of these immune checkpoint molecules.

### 3.6 Risk score-based stratification predicts the immune response and chemotherapy efficacy

**Figure 7:**
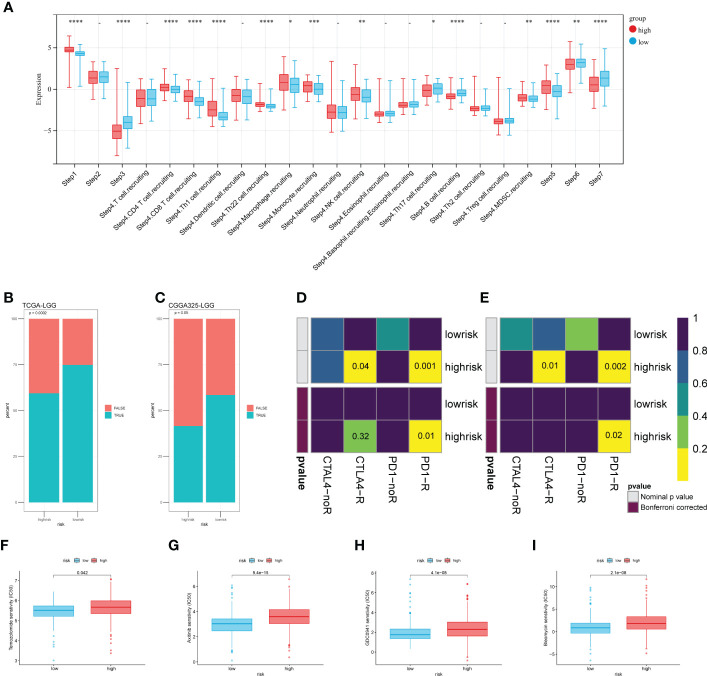
Risk score-based analysis of the stratifiable immune response and chemotherapy efficacy. **(A)** The TIP system quantified seven steps of the antitumor immune response. **(B, C)** Predicted response of TIDE to immune checkpoint inhibitors. **(D, E)** Comparing the effectiveness of PD1 and CTLA4 in response to different risk groups. **(F–I)** The sensibility of chemotherapeutic drugs **(F)** Temozolomide, **(G)** Axitinib, **(H)** GDC0941, and **(I)** Bleomycin. (*p < 0.05, **p <0.01, ***p <0.001, ****p <0.0001).

To explore the risk score stratification and the associated characteristics of the antitumor immune response, we introduced the Tracking Tumor Immunophenotype (TIP) system, which analyzed the status of anticancer immunity and the proportion of tumor-infiltrating immune cells across seven-step Cancer-Immunity Cycle. As shown in **Figure 7A**, the scores of the high-risk group were significantly higher in tumor antigen release (step 1), immune cell recruitment (step 4), and immune cell infiltration into the tumor (step 5). Conversely, the low-risk group scored significantly higher in T cell priming and activation (step 3), T cell recognition of cancer cells (step 6), and killing of cancer cells (step 7). Then, we used the CGGA dataset to validate these results (**Figure S3A**). Next, we introduce the TIDE algorithm to assess the efficacy of risk score in predicting ICB responsiveness. We found there was a significant difference in response to ICB treatment between the two groups (p< 0.001), and the response to ICB treatment was more sensitive in the low-risk group (**Figures 7B, C** **)**. SubMap was used to compare the prediction response to anti-PD1 and anti-CTLA4 therapy results with another dataset containing 47 patients with melanoma that responded to immunotherapies. Using this tool, we found that the high-risk group in the train and test sets showed comparable performance in predicting the LGGs’ response to anti-PD1 therapy (**Figures 7D, E** p< 0.05). Anti-CTLA4 therapy also showed a partial response in the TCGA train set (**Figure 7D** Bonferroni corrected p = 0.32).

We tried to analyze the response of two risk groups to chemo-drug efficacy. Here, in addition to TMZ, we selected three other chemo drugs from the literature that may have therapeutic potential for glioma. As expected, the sensitivity in the low-risk group was slightly better than that in the high-risk group (**Figure 7F**). That may be related to the level of MGMT promoter methylation in the low-risk group (**Figure 5E**). For the other three drugs, the estimated IC50 was significantly better in the low-risk group (**Figures 7G–I**). These results were validated using the CGGA325 dataset (**Figure S3B–E**). Although these drugs have not been used in clinical treatment on a large-scale, differences in sensitivity suggest that they have potential as novel therapeutic agents.

### 3.7 Genomic alterations of prognostic signature

**Figure 8:**
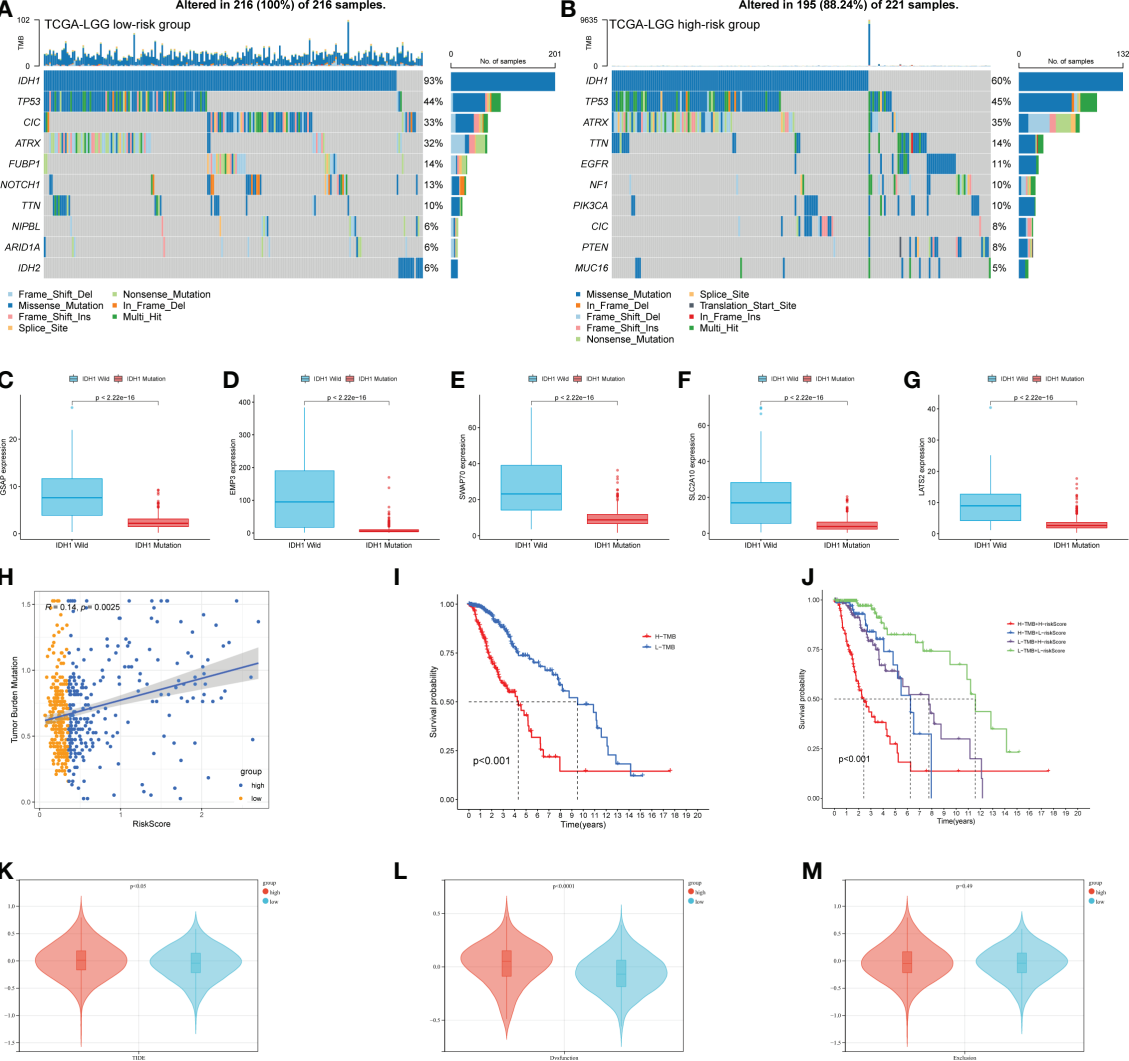
Epigenetic analysis of risk genes. **(A, B)** The mutation of high- and low-risk groups (Top 10 mutated genes). **(C–G)** Expression of 5 risk genes in the presence of different IDH1 mutations status. **(H)** Correlation analysis of risk score and TMB. **(I)** K-M curves of the high-TMB and low-TMB groups **(p< 0.001)**. **(J)**The combined risk score and TMB analysis of K-M curves in LGGs patients. **(K–M)** TIDE algorithm to model tumor immune evasion, **(K)** TIDE score, **(L)** Dysfunction score, and **(M)** Exclusion score.

Tumor genomic alterations have profound effects on immunity and drug therapy. We investigated the mutation frequencies of different risk groups and showed the top ten most frequently mutated genes (**Figures 8A, B**). As we expected, IDH1 had the highest mutation frequency, predominantly missense. Remarkably, some genes were associated with the immune microenvironment and immunotherapy. These genes were more prominent in the high-risk group, such as ATRX, EGFR, and PTEN. We targeted IDH1, the most frequently mutated of the two groups in our study and analyzed the relationship between the expression of five risk genes and IDH1 mutations. The results were shown in **Figures 8C–G**. The expression of all risk genes was higher in samples with wild-type IDH1 than in mutant IDH1. These risk genes and corresponding risk scores are consistent with the findings summarized in the preceding text (**Figure 5I**). In addition, we analyzed correlations between tumor mutational burden (TMB) and risk score. Like previous research, LGGs patients in the high TMB group have a poorer prognosis. Hence, we introduced the risk score and analyzed it jointly with TMB. Our results show that the risk score had a low positive correlation with TMB values (**Figure 8H**). Meanwhile, the high-risk group with a poorer prognosis corresponded to high TMB values (**Figure 8I**). Combining the two elements in the analysis, we found that LGGs samples with high-risk scores and high TMB values had the worst prognosis (**Figure 8J**). Finally, we compared the TIDE, dysfunction, and exclusion scores between the different risk groups. The TIDE score in patients with low-risk scores was significantly higher than those with high-risk scores (**Figure 8K** p< 0.01). In parallel, patients in the high-risk group had higher dysfunction scores constructed using dysregulated immune genes (**Figure 8L** p< 0.001). No difference was found between the two groups for exclusion scores constructed using immune rejection genes (**Figure 8M**). The mechanism of immune escape in these high-risk LGGs samples may be immune dysfunction rather than immune exclusion. These epigenetic alterations may affect the prognostic model for the therapeutic assessment of LGGs patients.

### 3.8 Experimental verification in cell lines and tissues

**Figure 9:**
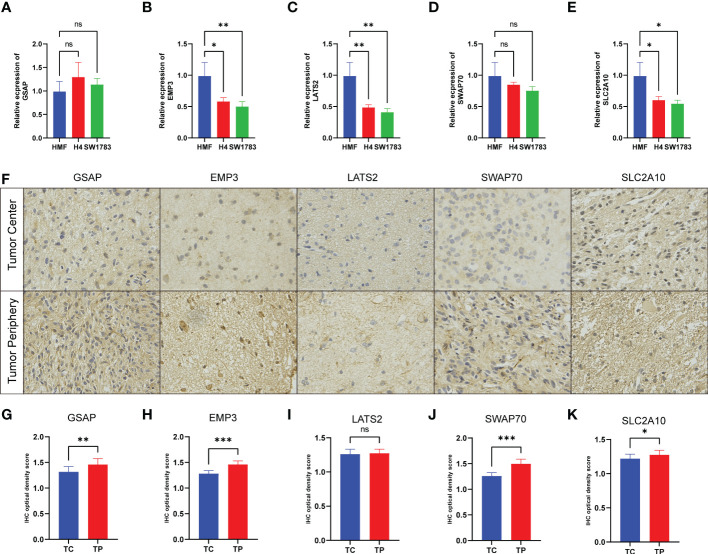
Experimental verification in cell lines and tissues. **(A–E)** The mRNA levels were determined by qRT-PCR in three cell lines. **(F)** The expression of five protein comparison of tumor center (TC) vs tumor periphery (TP) sites. **(G–K)** Bar graph showing the five protein levels obtained by quantification of immunohistochemical images. (*p<  0.05, **p < 0.01, and ***p < 0.001).

After obtaining the above five risk genes, we identified them at the cellular and protein levels. Given the rarity of fibroblasts in brain tissue, we chose a stable human mammary fibroblast (HMF) cell line as a control group. T98G and U251 cell lines are commonly used glioma cell lines for experiments. GSAP and SWAP70 expressed similar levels in different cell lines, and the expression of the remaining three genes was lower in glioma cell lines (**Figures 9A–E**). For protein expression, three patients were analyzed with IHC. LGGs tissues were obtained from the tumor center (TC) and tumor periphery (TP). We found the expression of five proteins was highly expressed in tumor periphery (**Figure 9F**). After a 4-step grading system was quantified, except for LATS2, other proteins showed high expression in the TP group (**Figures 9G–K**). This founding may be related to the research that tumor cells can influence the recruitment of CAFs precursors and induce the activation of normal fibroblasts into CAFs.

## 4 Discussion

Abnormal epigenetic alterations contribute to tumorigenesis and progression, as reflected in the latest guidelines for glioma classification (27). DNA methylation has been found to regulate microRNAs and predict overall survival in glioma (28, 29). Recurrence of LGGs occurs mainly within a few centimeters of the resected cavity, even in complete tumor resection and adjuvant chemotherapy (30, 31). Glioma recurrence and prognosis are closely related to alterations of TME (32, 33). Immune cells and CAFs are essential components of the TME. Here, we combined abnormal methylation and CAFs abundance for an in-depth analysis of LGGs, which is essential for a more comprehensive understanding of TME and developing stromal CAFs-targeted therapies.

Herein, we analyzed DMEGs in different CAFs abundance groups, and we found that the functional annotations of these DMEGs were enriched in pathways of tumorigenesis, progression, and malignant transformation. Compelling evidence shows that the extracellular matrix acts as the “soil” for malignant tumor progression and immune resistance (34). Among them, functions closely related to CAFs, such as the Wnt signaling pathway plays a vital role in the carcinogenic activity of LGGs (35). Oligodendrocyte differentiation reflects the stemness of glioma cells (36). EMT has been implicated in cancer stemness, invasiveness, and drug resistance (23). The genes with simultaneous alterations in gene expression and methylation levels may be factors that alter LGGs functions through CAFs. DNA methylation at these promoter regions is widely known to correlate negatively with gene expression levels (37). GO enrichment analysis revealed the main functions and hub genes involved in DMEGs. It is worth noting that in TSS200 and TSS1500 regions, MDK is a critical player in cancer progression and immune microenvironment (38). MMP14 regulates the activity of multiple extracellular and plasma membrane proteins, influencing cell-cell and cell-extracellular matrix communication (39).

After integrating clinical information, we constructed a prognostic signature based on five genes (EMP3, GSAP, LATS2, SLC2A10, and SWAP70). Compared to fibroblast cell lines and glioma cell lines using the CCLE database, we found that the expression of GSAP was similar. In the validation of *in vitro* cell experiments, in addition to GSAP, SWAP70 expression was also similar to fibroblasts. However, no evidence exists that any glioma cell line can represent LGGs or GBM. Its predictive value appears to be quite weak. In order to test protein expression, we performed sampling and IHC analysis in the center and periphery of LGGs. The expression of EMP3, GSAP, SLC2A10, and SWAP70 was higher in tumor periphery. This finding suggests that there may be more activated fibroblasts at the TP, and CAFs could function at tumor periphery. However, there is no significant difference in LATS2 expression between TC and TP. Despite LATS2 having been recognized as a target gene of CAFs-derived exosome microRNA-92 in breast cancer (40). SLC2A10 regulated fibroblasts in arterial tortuosity syndrome by encoding glucose transporter 10 (41). The PI3K-dependent recruitment of SWAP70 to the plasma membrane has been observed in growth factor-stimulated fibroblasts (42). EMP3 plays an important role in the regulation of membrane receptors associated with IDH-Wild type glioblastoma (43).

More and more research focused on the immuno-phenotype and immunotherapy of glioma cells. The high-risk scoring group showed increased antitumor immune cells macrophage M2 and M0. Despite glioma being defined as a cold tumor, proportions of macrophages can still constitute up to 30–50% of the TME (44–46). Surprisingly, mast cells activated were higher in the low-risk group. We quantified antitumor immunity in seven steps and further evaluated the antitumor immune process. Increased risk in the LGGs sample was accompanied by a decrease in the score of T cell priming and activation (step3) and destruction of tumor cells (step6, 7). It corresponds to a higher density of antitumor immune infiltration in the high-risk group. Immunotherapy, especially ICB, has brought paradigm shifts to cancer treatment. We found that the specific inhibitory immune checkpoints PD1 and CTLA4 were significantly overexpressed in the high-risk group (**Figure 6J**), while the Submap approach suggests that the high-risk group showed promising performance in predicting LGGs predicted response to anti-PD1 and anti-CTLA4 therapies **(** **Figures 7D, E** **)**. Other immune checkpoints that are highly expressed in high-risk groups are also being studied in an expanding way, such as the inhibitory immune checkpoints CD276 (47) and IL-10 (48), and the stimulatory checkpoints ICOS (49) and CD40 (50) are involved in the regulation of T cell function. TGFB1 can increase endothelial and epithelial permeability. It is more inclined to promote GBM cell invasion (51). SLAMF7 is a cell surface receptor involved in natural killer cell activation that received approval for treating multiple myeloma (52). Treatment by blocking or stimulating these new checkpoints in LGGs holds the promise of going beyond traditional PD1 therapies. Although our risk score distinguished the expression of ICPs of LGGs and predicted anti-ICB therapy, it still lacks elucidation of the interaction mechanism between CAFs and ICB. We hope to provide new ideas on the relationship between the treatment of immune checkpoints and CAFs.

TMZ has become the conventional chemotherapeutic agent for glioma however, TMZ resistance is the main factor that leads to current studies aimed at expanding multiple chemotherapeutic agents for glioma (53). Besides TMZ, three promising drugs were introduced in this study (**Figures 7G–I**). Axitinib induces senescence-associated cell death and necrosis in glioma (54). The PI3K inhibitor GDC-0941 enhances radiosensitization and reduces chemoresistance to temozolomide in GBM (55). Bleomycin inhibits proliferation and promotes apoptosis of glioma *via* the TGF-β/Smad signaling pathway (56). All three drugs showed promising IC50 in the low-risk group, and we hope this study will provide potential directions for the relationship between new chemotherapeutic agents and CAFs. However, these speculations are still at the level of data analysis, and whether these drugs can be applied to LGGs still needs a lot of experimental verification, such as molecular docking.

Solid evidence suggests that TMB plays a vital role in tumor immune escape (57). TP53 was frequently mutated in the high-risk subtype, and its mutation was reported to be associated with a poorer prognosis. Mutations in IDH1 characterize the majority of lower-grade gliomas in adults and define a subtype associated with a favorable prognosis (58, 59). Glioma shows a markedly elevated mutation burden, referred to as TMB-H (60). A study suggests that some gliomas with high TMB may benefit from PD-1 blockade therapy (61). Interestingly, MMR deficiency gliomas with TMB-H also lack significant inflammatory CD8+ infiltrates (62). On the other hand, TIDE algorithm analysis revealed that the mechanism of immune escape in LGGs samples might be immune dysfunction (**Figure 8L**). Even in the presence of a high level of infiltration by cytotoxic T cells, immune escape is still inevitable (63). However, it is worth noting that our study also has some limitations. Although our study identified five risk genes, they could not directly serve as CAFs marker genes in LGGs. We should further confirm the role of these CAFs risk genes on glioma cells using *in vitro* co-culture or single-cell multi-omics. Bioinformatics has always been used to research CAFs, but how gliomas induce the production of CAFs and exercise their functions in TME still requires extensive *in vivo* or *in vitro* experimental studies. These efforts will be included in our future studies.

## Data availability statement

The original contributions presented in the study are included in the article/**Supplementary Materials**. Further inquiries can be directed to the corresponding authors.

## Ethics statement

The studies involving human participants were reviewed and approved by The Animal Ethical and Welfare Committee of Zhejiang Provincial People’s Hospital. The patients/participants provided their written informed consent to participate in this study.

## Author contributions

SH, JWD, and HJ conceived and designed the study and revised the manuscript. JWD, HZ, JJ, XG, and XY provided analytical technical support. ZL, NW, and JZ participated in the production of charts and pictures. JWD and FW designed and completed qRT-PCR and IHC experiments. YB, JYD, and SM revised the manuscript. SH supervised the study. All authors contributed to the article and approved the submitted version.

## Supplementary material

Supplementary Figure 1 Clinical characteristics and risk scores in CGGA-693 LGGs test set. **(A)** K-M curves showed that the high-risk subgroup had worse overall survival than the subgroup in the test set **(**p< 0.001**)**. **(B)** K-M curves of different risk groups in WHO II and WHO III gliomas, **(C)** MGMT promoter status, **(D)** 1p19q codeletion status, **(E)** IDH mutation status, and **(F)** age effect.

Supplementary Figure 2 Overall survival analysis on immune checkpoint for LGGs patients. Grouping by median expression of immune checkpoint genes.

Supplementary Figure 3 Immunotherapy response and chemotherapy sensitivity in the CGGA. **(A)** TIP system analyzed the status of anticancer immunity and the proportion of tumor-infiltrating immune cells across the seven-step Cancer-Immunity Cycle. **(B)** Chemosensitivity of TMZ, p = 0.11. **(C)** Chemosensitivity of Axitinib, p< 0.001. **(D)** Chemosensitivity of GDC0941, p< 0.001. **(E)** Chemosensitivity of Bleomycin, p = 0.004.

Supplementary Table 1 The primers used in this study.

Supplementary Table 2 Clinical characteristics of patient cohort.

Supplementary Table 3 DEGs in TCGA.

Supplementary Table 4 DMGs in TSS200 region

Supplementary Table 5 DMGs in TSS1500 region

Supplementary Table 6 The formula of calculate risk score.
