# Supplementary figures and images for: Integrated analysis of genome-wide DNA methylation and cancer-associated fibroblasts identified prognostic biomarkers and immune checkpoint blockade in lower grade gliomas
Source: Front Oncol. 2023 Jan 16;12:977251. doi: 10.3389/fonc.2022.977251 (PMC9885112; doi:10.3389/fonc.2022.977251)

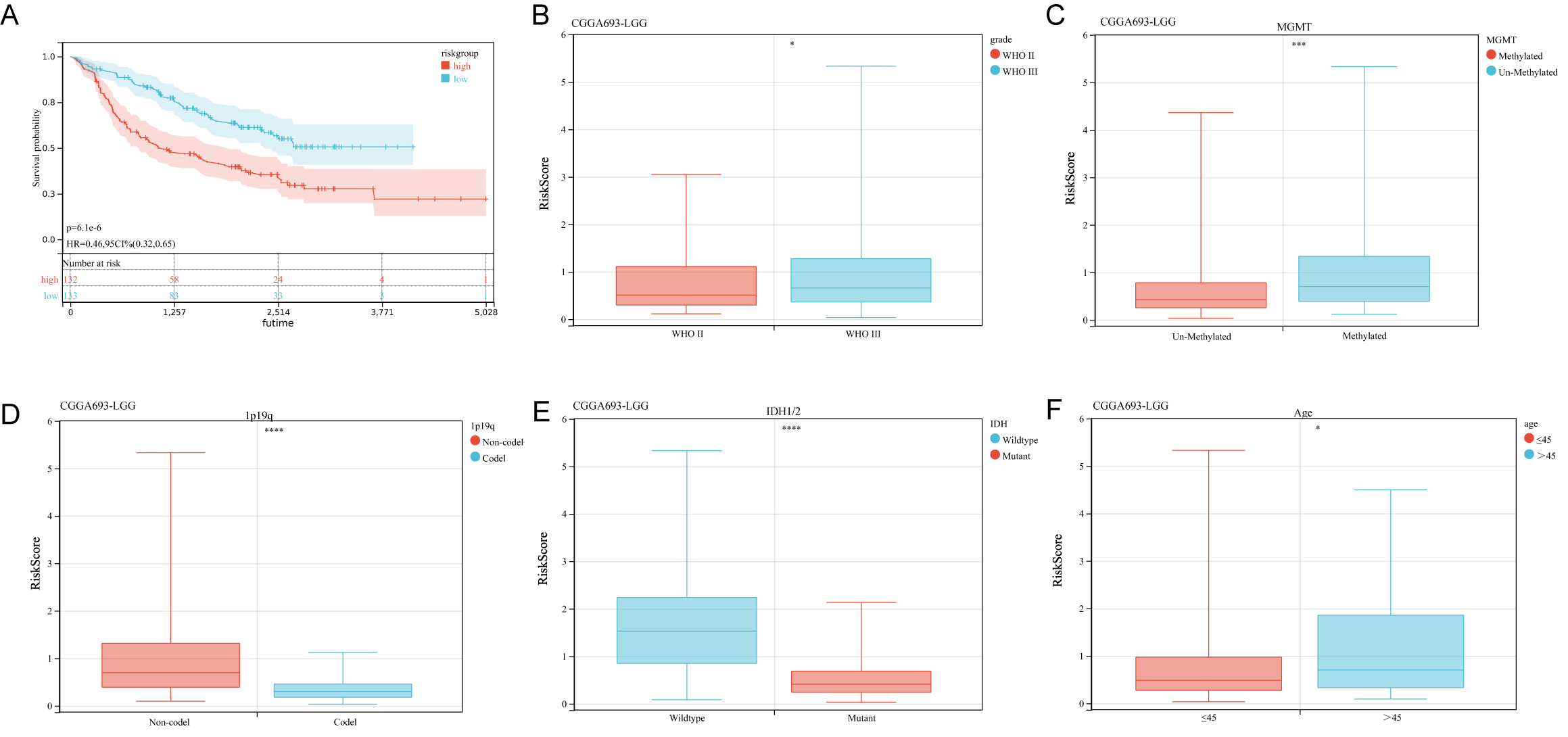

Supplement: Supplementary Figure 1 — Clinical characteristics and risk scores in CGGA-693 LGGs test set. (A) K-M curves showed that the high-risk subgroup had worse overall survival than the subgroup in the test set (p< 0.001). (B) K-M curves of different risk groups in WHO II and WHO III gliomas, (C) MGMT promoter status, (D) 1p19q codeletion status, (E) IDH mutation status, and (F) age effect. [file Image_1.tif]

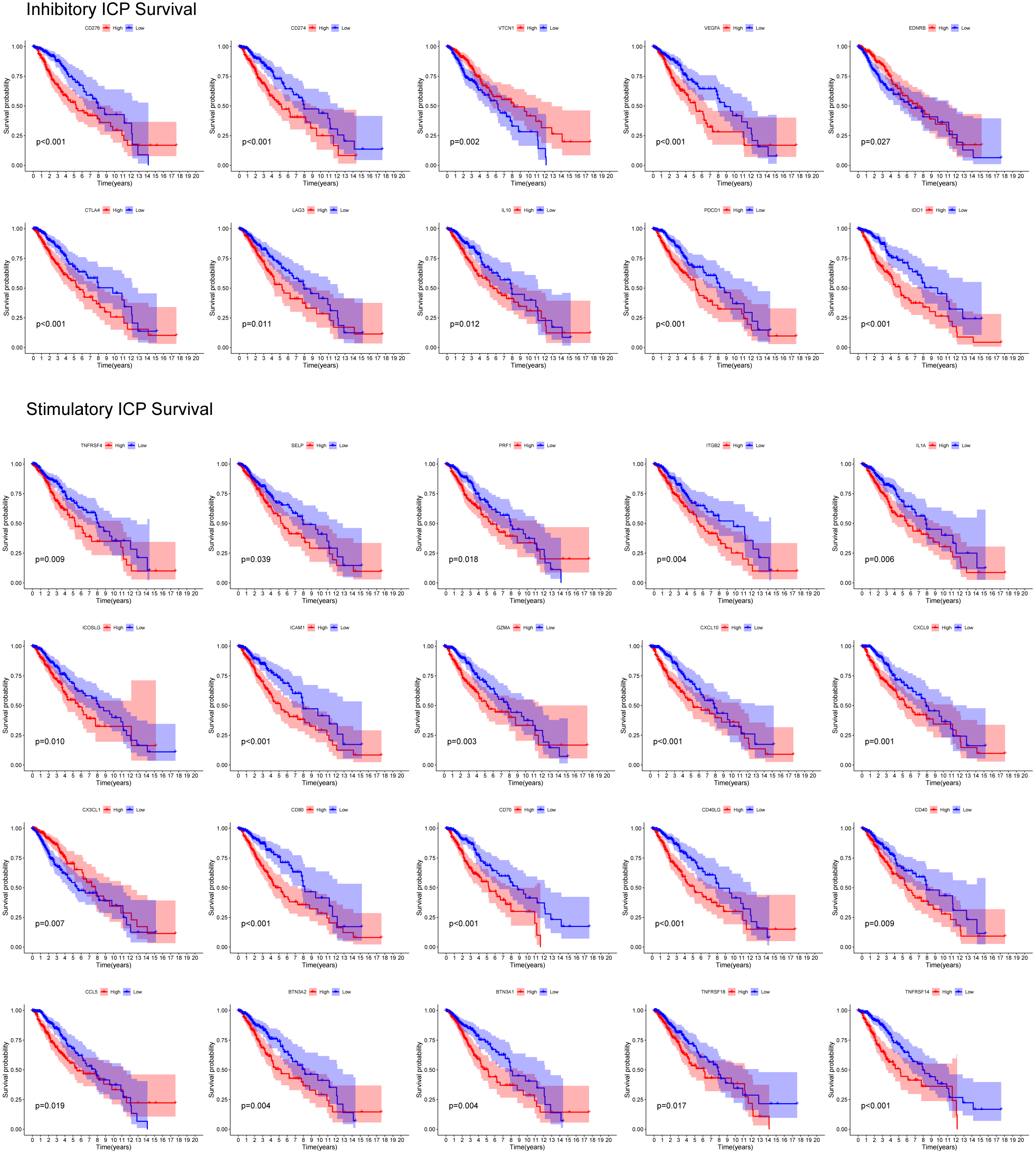

Supplement: Supplementary Figure 2 — Overall survival analysis on immune checkpoint for LGGs patients. Grouping by median expression of immune checkpoint genes. [file Image_2.tif]

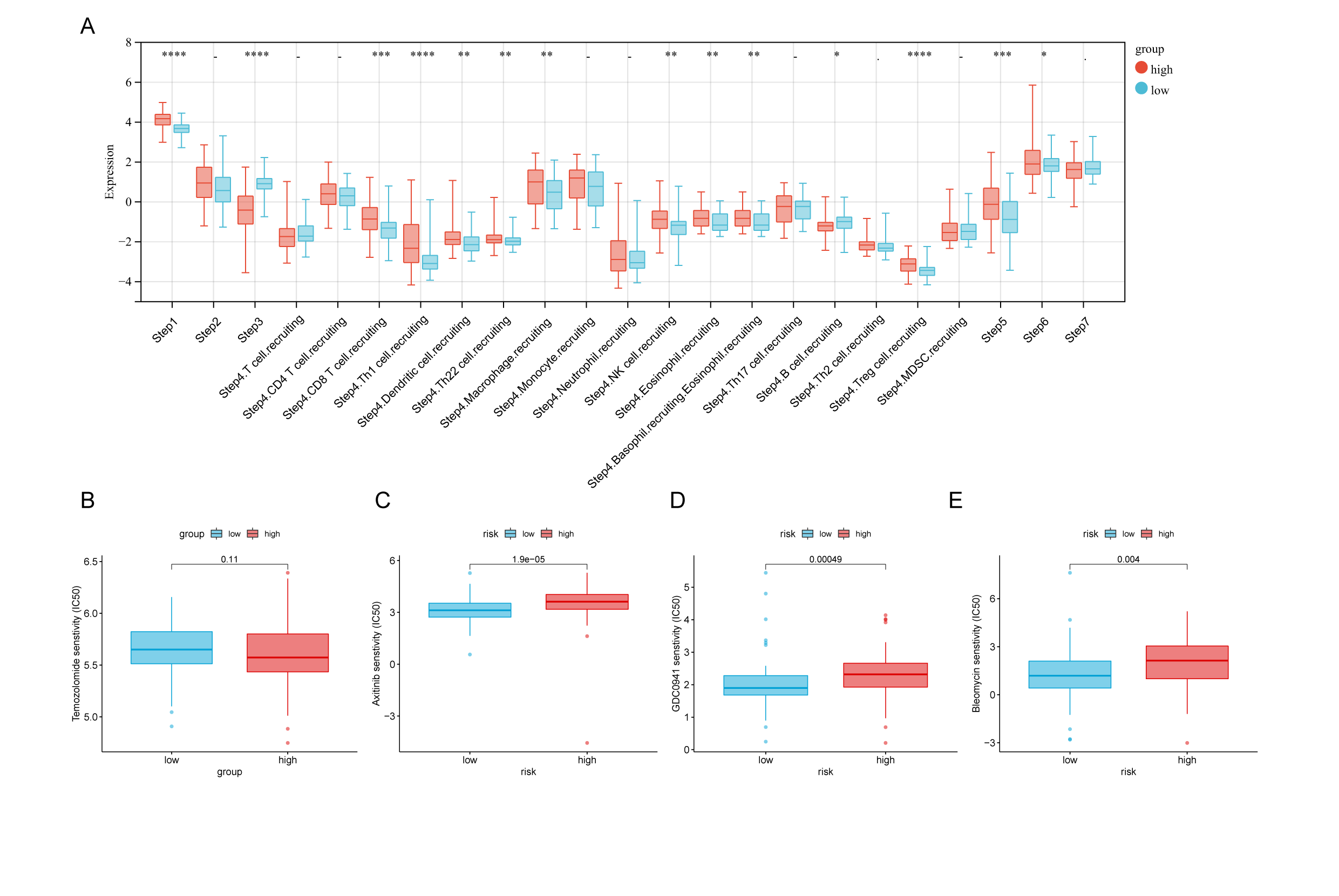

Supplement: Supplementary Figure 3 — Immunotherapy response and chemotherapy sensitivity in the CGGA. (A) TIP system analyzed the status of anticancer immunity and the proportion of tumor-infiltrating immune cells across the seven-step Cancer-Immunity Cycle. (B) Chemosensitivity of TMZ, p = 0.11. (C) Chemosensitivity of Axitinib, p< 0.001. (D) Chemosensitivity of GDC0941, p< 0.001. (E) Chemosensitivity of Bleomycin, p = 0.004. [file Image_3.tif]
