# Supplementary material for: Integrated analysis of genome-wide DNA methylation and cancer-associated fibroblasts identified prognostic biomarkers and immune checkpoint blockade in lower grade gliomas
Source: Front Oncol. 2023 Jan 16;12:977251. doi: 10.3389/fonc.2022.977251 (PMC9885112; doi:10.3389/fonc.2022.977251)
Supplement: Supplementary Table 1 — The primers used in this study. [file Table_1.docx]

Table S1

| id | name | 5'-3' |
| --- | --- | --- |
| 004P2022082900968 | EMP3-F | TGGGGTGTGTCAAGATGCTG |
| 004P2022082900969 | EMP3-R | CTGATGCACAATAGGCACGC |
| 004P2022082900970 | GSAP-F | GACTTCGACCTCGGGAAGGA |
| 004P2022082900971 | GSAP-R | GCTATCCACAGCCTTTAGAACC |
| 004P2022082900972 | LATS2-F | GAATGCCAACAATGTAGCGAATG |
| 004P2022082900973 | LATS2-R | GCCTCATTGTTAGTCCAGTTTCC |
| 004P2022082900974 | SWAP70-F | CCTCACAAAGAATCCCCTGCTC |
| 004P2022082900975 | SWAP70-R | GGCAAGGACTCTACACAGCAA |
| 004P2022082900976 | SLC2A10-F | TCATCCCCGCCTTTGAGACA |
| 004P2022082900977 | SLC2A10-R | GTGTGCTTGGGGTGTGCTAC |
