# Supplementary material for: Integrated analysis of genome-wide DNA methylation and cancer-associated fibroblasts identified prognostic biomarkers and immune checkpoint blockade in lower grade gliomas
Source: Front Oncol. 2023 Jan 16;12:977251. doi: 10.3389/fonc.2022.977251 (PMC9885112; doi:10.3389/fonc.2022.977251)
Supplement: Supplementary Table 6 — The formula of calculate risk score. [file DataSheet_1.docx]

RiskScore=0.00229154368328058*EMP3+0.0473175569810991*GSAP+0.00187506968152357*LATS2+0.000901343973389782*SLC2A10+0.01906597756407*SWAP70
